# PD-1 Blockade Reverses Obesity-Mediated T Cell Priming Impairment

**DOI:** 10.3389/fimmu.2020.590568

**Published:** 2020-10-29

**Authors:** Catherine T. Le, Lam T. Khuat, Sofia E. Caryotakis, Marilyn Wang, Cordelia Dunai, Alan V. Nguyen, Logan V. Vick, Kevin M. Stoffel, Bruce R. Blazar, Arta M. Monjazeb, William J. Murphy, Athena M. Soulika

**Affiliations:** ^1^ Department of Dermatology, School of Medicine, University of California, Davis, Sacramento, CA, United States; ^2^ Institute for Pediatric Regenerative Medicine, Shriners Hospitals for Children Northern California, Sacramento, CA, United States; ^3^ Department of Radiation-Oncology, School of Medicine, Comprehensive Cancer Center, University of California, Davis, Sacramento, CA, United States; ^4^ Masonic Cancer Center, and Division of Blood and Marrow Transplantation, Department of Pediatrics, University of Minnesota, Minneapolis, MN, United States; ^5^ Department of Internal Medicine, Division of Hematology and Oncology, School of Medicine, University of California, Davis, Sacramento, CA, United States

**Keywords:** T cell, priming, programmed cell death protein 1, dendritic cells, experimental autoimmune encephalomyelitis

## Abstract

Despite obesity reaching pandemic proportions, its impact on antigen-specific T cell responses is still unclear. We have recently demonstrated that obesity results in increased expression of PD-1 on T cells, and checkpoint blockade targeting PD-1/PD-L1 surprisingly resulted in greater clinical efficacy in cancer therapy. Adverse events associated with this therapy center around autoimmune reactions. In this study, we examined the impact of obesity on T cell priming and on autoimmune pathogenesis using the mouse model experimental autoimmune encephalomyelitis (EAE), which is mediated by autoreactive myelin-specific T cells generated after immunization. We observed that diet-induced obese (DIO) mice had a markedly delayed EAE onset and developed milder clinical symptoms compared to mice on control diet (CD). This delay was associated with impaired generation of myelin-specific T cell numbers and concurrently correlated with increased PD-L1 upregulation on antigen-presenting cells in secondary lymphoid organs. PD-1 blockade during the priming stage of EAE restored disease onset and severity and increased numbers of pathogenic CD4+ T cells in the central nervous system (CNS) of DIO mice to similar levels to those of CD mice. Administration of anti–PD-1 after onset of clinical symptoms did not increase EAE pathogenesis demonstrating that initial priming is the critical juncture affected by obesity. These findings demonstrate that obesity impairs antigen-specific T cell priming, but this can be reversed with PD-1 blockade. Our results further suggest that PD-1 blockade may increase the risk of autoimmune toxicities, particularly in obesity.

## Introduction

According to the CDC National Center for Health Statistics, approximately 40% of adults in the United States are overweight (defined as body mass index (BMI) 25 ≤ x < 30) or obese (BMI ≥ 30 kg/m^2^). Paradoxically, diet-induced obesity has been found to both promote and inhibit immune responses (1, 2). Increasing accumulation and activation of immune cells in adipose tissue promotes chronic low-grade inflammation (1), and obesity has been linked with autoimmune diseases, including inflammatory bowel disease (IBD), ankylosing spondylitis, multiple sclerosis (MS), and psoriasis (3, 4). Heightened inflammatory responses in obesity may in part be attributed to potentiation of pro-inflammatory macrophages at steady state (5, 6). Yet, obesity is also associated with increased immunosuppression, impaired innate immune cell function, and immune exhaustion, resulting in ineffective immune responses in infections and cancer (7–11). Furthermore, poorer vaccine responses have been correlated with increasing BMI (12, 13).

PD-1 is expressed on activated T cells, macrophages, dendritic cells, and B cells (14, 15). PD-1 regulates the naïve-to-effector transition and effector functions in T cells (16, 17), and binding to its ligand PD-L1 primarily suppresses CD28 signaling (18, 19). PD-L1 is ubiquitously expressed, while PD-L2 is predominantly expressed on antigen presenting cells (APC), and both ligands are upregulated during inflammation (14). Activation of the PD-1/PD-L1 pathway has been shown to inhibit autoimmunity, particularly in experimental autoimmune encephalomyelitis (EAE), an animal model of autoimmune-mediated neuroinflammatory disorders such as MS (20–25). Immunotherapy targeting the PD-1/PD-L1 pathway has gained FDA approval for several malignancies. While successful in the clinic, PD-1/PD-L1 blockade therapy must be closely monitored for immune-related adverse events, including autoimmunity (15).

One of the factors that dysregulate the PD-1/PD-L1 pathway is diet-induced obesity (DIO). In DIO mice, CD4+ T cells display increased senescence phenotypes with compromised proliferation, reduced IFN-γ production, and upregulation of PD-1 in visceral fat (26). DIO mice have increased numbers of PD-L1 expressing myeloid-derived suppressor cells (MDSCs) in the tumor microenvironment, and global PD-L1-deficiency increases survival in the mammary carcinoma model 4T1 in obese mice (27). Obesity drives PD-1-mediated T cell dysfunction and exhaustion, at least in part *via* a leptin-dependent mechanism, and increases responsiveness to anti–PD-1 immunotherapy (11). High BMI in cancer patients receiving PD-1/PD-L1 checkpoint blockade alone or in combination with other therapies has been associated with greater response and survival (11, 28). While these studies have highlighted an important impact of obesity on memory T cell biology through PD-1/PD-L1 regulation, it is unclear how this relationship impacts naïve T cell response to foreign or self-antigens.

In this study, we examined the impact of obesity on the PD-1/PD-L1 regulation of autoimmunity. Previous studies reported that high-fat diets augment EAE severity (29, 30). Surprisingly, our results show that DIO mice fed high-fat diet for prolonged periods of time had markedly delayed onset and reduced severity of EAE clinical symptoms compared to control mice. This was correlated with impairments in myelin-specific T cell generation in secondary lymphoid organs (SLO) and higher levels of dendritic cells (DCs) expressing PD-L1 in obese mice compared to their controls after immunization. Antigen-specific T cell priming in response to either autoreactive or xenogeneic antigens was blunted in the obese environment compared to control mice. Administration of PD-1 blockade during the priming phase effectively increased antigen-specific T cell numbers and restored EAE severity in obese mice to levels comparable to those of controls. This indicates that PD-1 blockade is a good strategy to restore T cell responses in obesity with the possible caveat of increased risk for autoimmunity.

## Materials and Methods

### Mice

Male C57BL/6NTac aged 5–6 weeks were purchased from Taconic Farms and housed in a specific pathogen-free facility. Diet-induced obese (DIO) and control mice were fed open-source purified diet consisting of either 60% fat or 10% fat (D12492 & D12450J, Research Diets, Inc.), respectively, starting at 6-7 weeks of age.

### Experimental Autoimmune Encephalomyelitis

Mice were induced with EAE at indicated age by subcutaneous flank administration of 300 µg of rodent MOG peptide (amino acids 35-55, MEVGWYRSPFSRVVHLYRNGK, New England Peptide, Inc.) in Complete Freund’s Adjuvant (CFA) containing 5 mg/ml killed *Mycobacterium tuberculosis* H37Ra (Difco, Cat # 231141) in Incomplete Freund’s Adjuvant (Difco, Cat # 263910) on day 0 with intraperitoneal (IP) administration of 200 ng of *Bordetella pertussis* toxin (List Biological Laboratories, Inc., Product # 180) on days 0 and 2 (31). The mice were weighed and examined daily. Clinical scores were monitored as described in (31) with modifications: no detectable signs = 0; distal limp tail = 0.5; limp tail or waddling gait = 1.0; limp tail and waddling gait = 1.5; single limb paresis = 2.0; double limb paresis = 3.0; severe double limb paresis = 3.25; single limb paralysis and paresis of second limb = 3.5; full paralysis of 2 limbs = 4; moribund = 4.5; death = 5; (31). PD-1 blockade (29F.1A12, Cat # BE0273, BioXCell) was administered on indicated days with 500 µg IP on the initial day and 250 µg IP every other day for total of six doses (11).

### Single Cell Suspension Protocol

Secondary lymphoid organs were harvested prior to whole body perfusion with ice-cold PBS. Spleens or draining lymph nodes were harvested, mechanically dissociated, and strained through 100-µm mesh. Red blood cells were lysed with ACK solution (BioLegend, Cat # 420301). Brains and spinal cords were collected after whole body perfusion with ice-cold PBS and minced, and then incubated at 37°C for 40 min in complete RF10c media containing 0.5 mg/ml Collagenase Type IV (Worthington Biochemical Corporation, Cat # LS004188) and 20 µg/ml of DNAse I (Worthington Biochemical Corporation, Cat # LS002139). Digested fragments were mechanically pushed through 100-µm mesh. Mononuclear cells were isolated from CNS with discontinuous 40/70% Percoll™ gradient (GE Healthcare, Cat # 17-0891-01) (31).

### MOG Peptide Recall Response

Splenocytes were plated at 250,000 cells/well in 12 wells in round-bottom 96 well plate and were cultured in 200 µl of complete RF10c media containing 10% Nu Serum (Corning IV Culture, Cat # 355504), 2 mM glutamine (Gibco, Cat # 25030-081), 1% nonessential amino acids (Corning, Cat # 25-025-CI), 1% penicillin-streptomycin (Corning, Cat # 30-002-CI), 5 × 10^−5^M 2-mercaptoethanol (Sigma-Aldrich, Cat # M7522-100), 1mM HEPES buffer (Gibco, Cat # 15630-080), 1 mM sodium pyruvate (Corning, Cat # 25-000-CI) with or without 50 µg/ml MOG peptide (amino acids 35-55, MEVGWYRSPFSRVVHLYRNGK, New England Peptide, Inc.) for 48 h at 37°C with 5% CO_2_. The cells were incubated at 37°C with 4 µl/6 ml GolgiStop (BD Biosciences) and 1 µl/ml GolgiPlug (BD Biosciences) for the last 5 h before surface staining and intracellular cytokine staining.

### Peptide Immunization

Mice were immunized by subcutaneous flank administration of 100 µg of OVA257-264 peptide (SIINFEKL, ThermoFisher Scientific) in Complete Freund’s Adjuvant (CFA) containing 1 mg/ml or 5 mg/ml killed *Mycobacterium tuberculosis* H37Ra (Difco, Cat # 231141) in Incomplete Freund’s Adjuvant (Difco, Cat # 263910) on day 0. The mice were weighed and examined every 2 days.

### Flow Cytometry

Single-cell suspensions were prepared from central nervous system, spleens, and draining lymph nodes. Cells were incubated with Fc block (anti-CD16/32 clone 93, BioLegend) and stained with the fluorochrome-conjugated monoclonal antibodies listed below. For intracellular staining, cells were incubated for at 37°C with 0.67 µl/ml GolgiStop (BD Biosciences) and 1 µl/ml GolgiPlug (BD Biosciences) for the last 3 h before surface staining. Intracellular cytokine staining of IFN-γ, IL-17α, and TNF was performed using Cytofix/Cytoperm™ kit (BD Biosciences). For intracellular staining of FoxP3, the FoxP3/Transcription Factor Staining kit (ThermoFisher Scientific) was used.

### Tetramer Staining

NIH Tetramer Core Facility (Emory University, Atlanta, GA) provided the following tetramers: APC-MHC class II tetramer, consisting of murine I-Ab complexed to GWYRSPFSRVVH (MOG38-49) peptide, APC-MHC class II tetramer, consisting of I-Ab complexed to PVSKMRMATPLLMQA (hCLIP) peptide, and PE-MHC class I tetramer, consisting of murine H-2Kb complexed to SIINFEKL (OVA257-264) peptide. MOG38-49 tetramer and hCLIP tetramer were stained at room temperature for 2 h prior to cell surface stain. M45 tetramer was stained at 4°C for 30 min with cell surface stain. OVA257-264 tetramer was stained at 4°C for 1 h prior to cell surface stain. All tetramers were stained at 1:100.

### Immunohistochemistry

Spinal cords were stained as previously published (32). Spinal cords were collected after whole body perfusion with ice-cold PBS and then post-fixed in 4% paraformaldehyde (PFA) (Electron Microscopy Sciences, Cat # 15710-S) in PBS for 24 h, then moved to 30% sucrose (Fisher Scientific, Cat # S5-500) in PBS for 72 h. Spinal cords were embedded in cryostat mounting media (Tissue-Tek OCT, Sakura Finetek). Ten-micrometer sections were dried at room temperature for 1 h, rinsed with PBS, and blocked with 10% donkey serum or goat serum (Jackson ImmunoResearch Laboratories, Inc., West Grove, PA), depending on the secondary antibody used. Primary antibodies were applied overnight at 4°C and were rat anti-mouse CD4 (Cat # 550280, BD Pharmingen, 1:100), rabbit polyclonal Iba-1 (Cat # 019-19741, Wako Chemicals, 1:500), rat anti-myelin basic protein (Cat # NB600-717, Novus Biologicals, 1:200), and mouse anti-Neurofilament H, nonphosphorylated (Cat # 801701, Biolegend, 1:500). Fluorescently conjugated secondary antibodies to DyLight™488 or DyLight™549 (Jackson ImmunoResearch, Inc, 1:500) or AlexaFluor-488, AlexaFluor-549, or Cyanine Cy5 (Jackson ImmunoResearch Laboratories, 1:500) for 1 h at RT. Nuclei were counter-stained with DAPI. Images were obtained by laser scanning confocal microscopy with 20× objectives (Nikon C2) and analyzed with the Nikon NIS-Elements software (Version 5.02).

### Quantitative Polymerase Chain Reaction (q-PCR)

Spinal cord segments at the L4-L5 lumbar section were stored in RNA*later*™ solution (ThermoFisher Scientific, Cat # AM7021), and RNA was isolated using RNeasy Lipid Tissue Mini Kit (Cat # 74804, Qiagen, Valencia, CA). Single cell suspension of pan-naïve T cells sorted by immunomagnetic negative selection following manufacturer’s instructions for EasySep Mouse Pan-Naïve T cell Isolation Kit (Cat # 19848, STEMCELL™ Technologies, Inc.) were snap frozen in liquid nitrogen, and RNA was isolated using RNeasy Mini kit (Cat # 74106, Qiagen). DNA was prepared using iScript™ cDNA Synthesis Kit (Cat # 1708891, Bio-Rad). qPCR was performed on CFX384 Touch™ Real-Time PCR Detection System (Bio-Rad) with SsoAdvanced Universal SYBR Green Supermix (Cat # 172-5271, Bio-Rad). The following primers were used for analysis: mouse IFN-γ (PPM03121A-200, Qiagen), mouse IL-17α (PPM03023A-200, Qiagen), mouse SOCS1 (qMmuCED0049519, Bio-Rad), mouse SOCS3 (qMmuCED0003480, Bio-Rad), and mouse GAPDH (PPM02946E-200, Qiagen). Data was analyzed on CFX Maestro™ Software (Bio-Rad).

### Cytometric Bead Array (CBA) Measurement

Serum cytokines were measured by CBA flex set kits (BD Biosciences): mouse IL-10 (Cat # 558300), mouse TNF (Cat # 558299), mouse IL-6 (Cat # 558301), GM-CSF (Cat # 558347), G-CSF (Cat # 560152), and KC (Cat # 558340). Serum samples were diluted 1:4, and manufacturer’s instructions were followed. Cytokine concentration was measured by LSR Fortessa flow cytometer (BD Biosciences) and analyzed using FlowJo 10.4.1 software (FlowJo, LLC).

### Colorimetric MTT Assay

Splenocytes were plated at 200,000 cells per well for 72 h with dose response of Concanavalin A (ConA, MilliporeSigma) at 37°C at 5% CO_2_ in 200 µl of complete RF10c media containing 10% Nu Serum (Corning IV Culture, Cat # 355504), 2 mM glutamine (Gibco, Cat # 25030-081), 1% nonessential amino acids (Corning, Cat # 25-025-CI), 1% penicillin-streptomycin (Corning, Cat # 30-002-CI), 5 × 10^−5^M 2-mercaptoethanol (Sigma-Aldrich, Cat # M7522-100), 1M HEPES buffer (Gibco, Cat # 15630-080), 1 mM sodium pyruvate (Corning, Cat # 25-000-CI). Proliferation was measured using Cell Proliferation Kit I MTT (Roche, Cat # 11 465 007 001), following manufacturer’s instructions. VerMax™ Tunable Microplate Reader was used to measure absorbance reading at 570 and 700 nm.

### MRI Imaging

Mice were anesthetized with isofluorane and oxygen and were scanned on the Biospec 70/30 7.0 Tesla small-animal MRI system (Bruker Biospin Inc) using a 60-mm quadrature transmitter/receiver coil for whole-body imaging with the assistance of UC Davis Center for Molecular and Genomic Imaging, as previously published (11).

### Data Analysis and Statistics

Graphs were made and statistical analyses were performed using GraphPad Prism Version 6.02 (GraphPad Software, Inc.). Data were expressed as mean ± S.D. or S.E.M, as indicated. One-way or two-way analysis of variance (ANOVA) tests were performed with Tukey post-hoc test or Holm-Sidak multiple comparison testing, as appropriate. Two-tailed Student’s t-test was used to compare differences between two normally distributed test groups. Mann-Whitney ranking U test was used to compare differences in EAE clinical scores. P values were considered statistically significant if p < 0.05. Statistical outliers were identified using ROUT test.

#### Study Approval

All experimental protocols were approved by the Institutional Animal Care and Use Committee of the University of California, Davis.

## Results

### Diet-Induced Obesity Alleviates Clinical Severity in EAE

**Figure 1:**
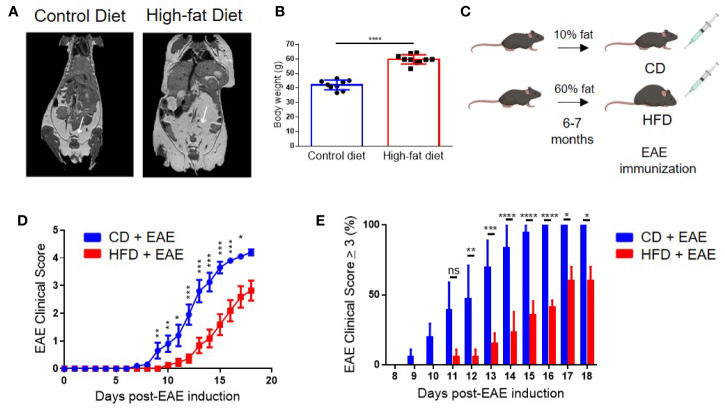
Diet-induced obesity delays the onset and dampens the severity of clinical symptoms in Experimental Autoimmune Encephalomyelitis (EAE). **(A)** MRI imaging of 6-month-old male control and DIO mice. White arrows point to adipose tissue. **(B)** Body weight of 8-month-old control and DIO mice. **(C)** Schema of experimental design. C57BL/6 male mice were placed on 10% fat (CD) or 60% fat diet (HFD) for 6-7 months and induced with EAE. **(D)** EAE clinical score. **(E)** Percent of mice with EAE clinical scores of severity of at least 3 per day. n = 13–14/group; means from three separate experiments. Bar graphs depict mean ± SEM. Significance for differences in clinical scores were determine by Mann-Whitney ranking U test. ns: not significant, *p < 0.05, **p < 0.01, ***p < 0.001, ****p < 0.0001.

C57BL/6 mice were placed on 60% fat diet (high-fat diet; HFD) or 10% fat diet (control diet; CD) starting at 6-8 weeks of age. After 6 months on the diet, DIO mice had more subcutaneous and visceral adipose tissue compared to the control mice placed on CD as visualized by magnetic resonance imaging (MRI) (**Figure 1A**) (11) and weighed statistically significantly greater than CD mice (p < 0.0001) (**Figure 1B**). At this age, we found no differences between DIO and CD mice in non-fasting glucose levels or differences in the percentages of glycated hemoglobin (Hb), as measured by the A1C test (**Supplemental Figure 1A**). It was previously shown that HbA1c percentage is similar between control and DIO mice after 4 or 9 months of HFD (11). Although hyperglycemia was previously observed in DIO mice at 4 months of HFD, this difference was lost after 9 months of HFD (11).

EAE was induced in 8- to 9-month-old (age-equivalent to a 35-year-old human) DIO and CD mice *via* immunization with MOG35-55 peptide in complete Freund’s adjuvant (CFA) (**Figure 1C**), which commonly results in 100% incidence of bilateral hindlimb paralysis (score ≥ 4) in 8- to 12-week-old mice (31, 33). Onset of clinical disease in CD mice was on day 9 post-EAE induction (p.i.) and peaked on day 16–18 p.i. with 100% of mice having a score of 3 or more (**Figures 1D, E** and **Supplemental Figure 1B**). In contrast, DIO mice had both delayed onset (day 12 p.i.) and milder neurological deficits, with only 57% developing a score of 3 or more by day 18 p.i. (**Figures 1D, E** and **Supplemental Figure 1B**). All DIO mice survived to day 20 p.i. compared to 85% of CD mice (**Supplemental Figure 1C**). Similar EAE clinical course was observed with different immunization protocols, varying the amounts of MOG peptide (**Supplemental Figure 1D**) or of the adjuvant (**Supplemental Figure 1E**). As the majority of EAE models utilize 8- to 16-week-old mice, we examined the impact of age on EAE clinical course using our standard immunization protocol. Control 8-month-old mice had comparable EAE clinical course with that of 8-week old mice (**Supplemental Figure 1F**). Additionally, mice placed on HFD for only 10 weeks still had impaired EAE clinical course compared to controls (**Supplemental Figure 1G**). However, the effect was milder than that observed after 6 month in HFD, and thus we continued the study using mice in long-term HFD. These results demonstrate that obesity impairs EAE induction and severity.

### Milder EAE Clinical Disease in DIO Mice Is Associated With Impaired CNS Infiltration by Peripheral Immune Cells

**Figure 2:**
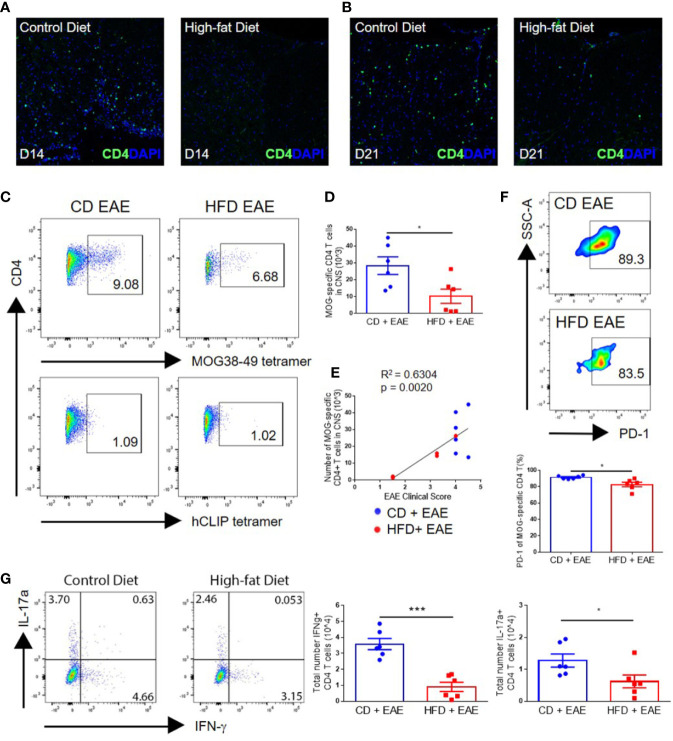
Impaired EAE clinical course in diet-induced obesity is associated with decreased numbers of antigen-specific T cells in the CNS. **(A–G)** C57BL/6 male mice were placed on CD or HFD for 6–7 months and induced with EAE. **(A, B)** IHC staining of CD4+ T cells in the spinal cord on day 14 and day 21 post-immunization in control mice and DIO mice. **(C)** Representative flow plots of MOG38-49 tetramer staining of CD4+ T cells in CNS. **(D)** Numbers of MOG38-49 tetramer+ CD4+ T cells in CNS on day 18 post-immunization. **(E)** Numbers of MOG38-49 tetramer+ CD4+ T cells in CNS versus EAE clinical score on day 18 post-immunization. **(F)** Representative flow plots and percentage of MOG-specific CD4+ T cells expressing PD-1. **(G)** Representative flow plots of IFN-γ and IL-17α expression in CD4+ T cells in the CNS. Numbers of IFN-γ+ CD4+ T cells and IL-17α+ CD4+ T cells in the CNS on day 18 p.i. Bar graphs depict mean ± SEM. Sample size n = 6/group from two separate experiments. Two-tailed unpaired Student’s t-test used to compare two groups. *p < 0.05, ***p < 0.001.

As EAE is mediated by myelin-specific CD4+ T cells, we assessed the levels of CNS infiltration by T cells at various time points following immunization. Greater numbers of CD4+ T cells were detected by immunohistochemistry in the spinal cord perivascular and subpial spaces, with greater penetrance into the parenchyma on days 14 and 21 p.i. in CD mice compared with DIO mice (**Figures 2A, B**). This was further supported by flow cytometric analysis using MOG-specific tetramer, demonstrating statistically significantly greater numbers of MOG38-49 specific CD4+ T cells in the CNS of control mice at the peak of the disease (day 18 p.i.) compared with DIO mice (28.4 × 10^3^ ± 12.8 vs. 10.2 × 10^3^ ± 10.3; p = 0.0233) (**Figures 2C, D**). Total numbers of MOG38-49-specific T cells in the CNS correlated with EAE clinical score (**Figure 2E**), demonstrating that impairments in the EAE course were due to reduced infiltration or activity of antigen-specific T cells in DIO mice. The majority of MOG-specific T cells in the CNS were PD-1 positive, indicating recent antigen activation, and CD mice had a greater percentage of MOG-specific T cells expressing PD-1 (91.4% ± 2.1 vs. 82.7% ± 6.7; p = 0.0127) (**Figure 2F**). Furthermore, greater numbers of IFN-γ-expressing CD4+ T cells (Th1) (3.6 × 10^4^ ± 0.9 vs. 0.9 × 10^4^ ± 0.7; p = 0.0002) and IL-17α-expressing CD4+ T cells (Th17) (1.3 × 10^4^ ± 0.5 vs. 0.6 × 10^4^ ± 0.5; p = 0.0451) were detected in the CNS of CD versus DIO mice (**Figure 2G**). In support of this, spinal cords of DIO mice had markedly reduced levels of *IFNG* and *IL17A* transcripts compared to those of CD mice on day 14 p.i. (**Supplemental Figure 2**). The observed increase of these transcripts in DIO mice on day 21 is likely reflective of the delayed EAE clinical onset in this group.

**Figure 3:**
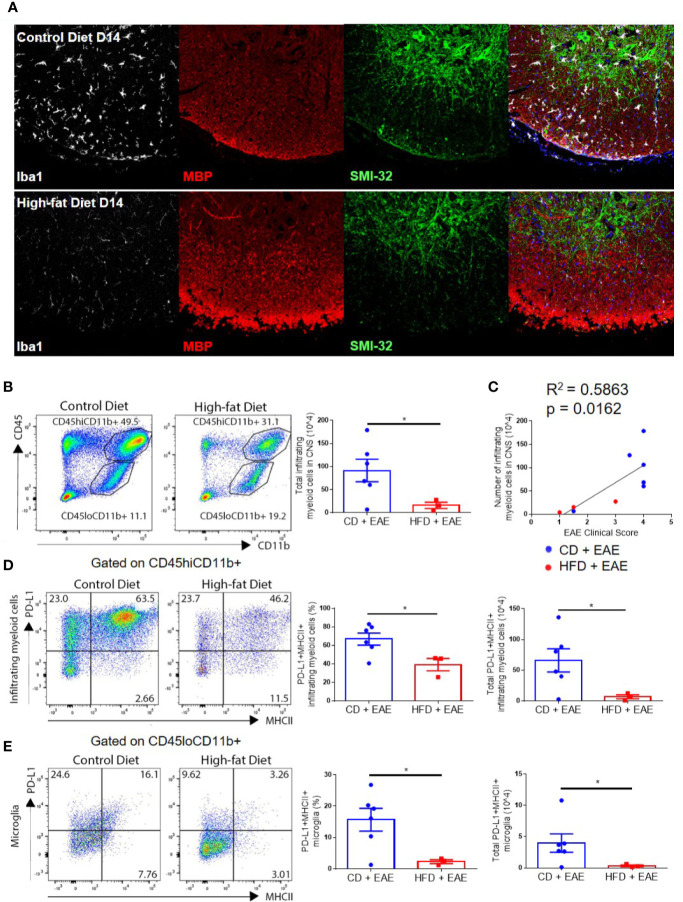
Numbers of infiltrating myeloid cells and PD-L1 upregulation correlate with EAE disease severity. **(A–E)** C57BL/6 male mice were placed on CD or HFD for 6–7 months induced with EAE. **(A)** Iba1 (white), myelin basic protein (red), and SMI-32 (green) immunoreactivity in spinal cords isolated from CD or DIO mice on day 14 post-immunization. **(B)** Representative flow plots and gating strategy for infiltrating myeloid cells (CD45hiCD11b+) and microglial cells (CD45loCD11b+) in the CNS on day 16 post-immunization. Total numbers of infiltrating myeloid cells in the CNS on day 16 post-immunization. **(C)** Numbers of infiltrating myeloid cells (CD45hiCD11b+) cells in CNS vs. EAE clinical score on day 16 post-immunization. **(D)** Representative flow plots indicating expression of PD-L1 and MHCII on infiltrating myeloid cells (left panels), percentage of PD-L1+MHCII+ infiltrating myeloid cells (middle panels), and total number of PD-L1+MHCII+ infiltrating myeloid cells (right panels) in the CNS on day 16 p.i. **(E)** Representative flow plots indicating expression of PD-L1 and MHCII on microglia (left panels), percentage of PD-L1+MHCII+ microglia (middle panels), and total number of PD-L1+MHCII+ microglia (right panels) in the CNS on day 16 p.i. Sample size n = 3–6/group; combined from two different experiments. Bar graphs depict mean ± SEM. Two-tailed unpaired Student’s t-test used to compare two groups. *p < 0.05.

CNS infiltration by monocyte-derived cells results in demyelination and axonal damage, marked by loss of myelin basic protein (MBP) staining and accumulation of hypo-phosphorylated neurofilament–H protein (detected by immunoreactivity for the antibody SMI-32) in the white matter, respectively (31). Both demyelination and axonal degeneration promote the progression of neurological deficits in EAE (31, 33–36). Immunohistological analysis on day 14 p.i. showed that DIO mice had little or no accumulation of infiltrating myeloid cells in the white matter and normally myelinated/undamaged axons, while CD mice had an apparent increase in IBA1 immunoreactive cells (microglia, as well as peripheral monocyte-derived cells), decreased MBP and increased SMI-32 immunoreactivity in the white matter (**Figure 3A** and **Supplemental Figure 3A**). Microglia in the gray matter (far from the lesion site) of CD mice also displayed activated morphology with amoeboid, fattened cell bodies and shortened processes, while gray matter microglia in the DIO mice appeared resting as indicated by their ramified morphology with small cell bodies and long, thin processes (**Supplemental Figure 3B**). By day 21 p.i., DIO mice had varying degrees of EAE severity with clinical scores correlating with the numbers of resident and infiltrating myeloid cells in the spinal cord, microglial activation, and apparent degree of axonal damage (**Supplemental Figures 3C, D**). Flow cytometry analysis also demonstrated statistically significantly greater numbers of infiltrating myeloid cells (CD45hiCD11b+) in the CNS of CD mice compared to DIO mice on day 16 p.i. (p = 0.0257) (**Figure 3B**). Similarly to T cells, the numbers of infiltrating myeloid cells were correlated with EAE clinical severity (**Figure 3C**). These results demonstrate that the CNS of DIO mice had reduced numbers of infiltrating cells and cytokine production correlating with the reduced disease induction.

### Decreased Inflammation in the CNS of DIO Mice Is Not Due to PD-L1 Upregulation on Microglia and CNS Infiltrating Myeloid Cells

PD-L1 upregulation on microglia and/or infiltrating myeloid cells in the CNS inhibits the function of myelin-specific CD4+ T cells in EAE (21, 22, 24, 37). Given recent evidence of PD-L1 upregulation in obesity (27), we speculated that decreased CD4+ T cell numbers within the CNS of DIO mice with EAE may reflect upregulation of PD-L1 in CNS resident or infiltrating myeloid cells. Interestingly, the numbers of PD-L1-positive infiltrating myeloid cells (66.1 × 10^4^ ± 46.2 vs. 6.9 × 10^4^ ± 5.7; p = 0.0340) and microglia (CD45loCD11b+) (4.0 × 10^4^ ± 3.6 vs. 0.3 × 10^4^ ± 0.2; p = 0.0401) were greater in CD mice on day 16 p.i. compared to DIO mice, reflecting that PD-L1 upregulation is correlated with activation status and disease progression (**Figures 3D, E**). This indicates that the reduced pathology in the CNS of DIO mice is not due to PD-L1 upregulation in the CNS.

### Impaired Antigen-Specific T Cells Priming in DIO Mice

**Figure 4:**
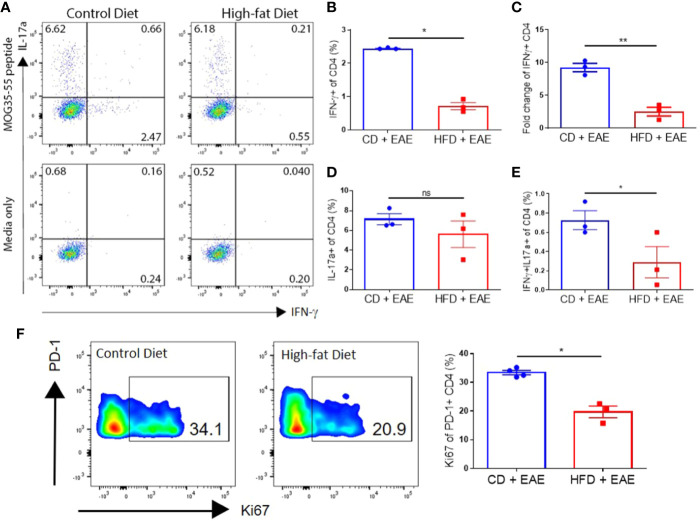
DIO mice have reduced MOG-specific T cells in secondary lymphoid organs after immunization. **(A–F)** C57BL/6 male mice were placed on CD or HFD for 6–7 months and induced with EAE. SLOs were harvested on day 8 p.i. **(A)** Representative flow plots of IFN-γ+ and IL-17α+ expressing by CD4+ T cells pulsed with or without 50 μg/ml MOG peptide for 48 h (MOG peptide recall assay). **(B)** Percentages of CD4+ T cells expressing IFN-γ (Th1) in MOG peptide recall assay. **(C)** Fold change in IFN-γ expression with MOG peptide pulse over media. **(D)** Percentages of CD4+ T cells expressing IL-17α (Th17) in MOG peptide recall assay. **(E)** Percentages of CD4+ T cells expressing both IFN-γ- and IL-17α (Th1/Th17) in MOG peptide recall assay. **(F)** Representative flow plots and quantification of percentage of PD-1+ CD4+ T cells expressing Ki67. Sample size n = 3–4/group; representative of two independent experiments. Bar graphs depict mean ± SEM. One-way analysis of variance (ANOVA) with Tukey’s *post hoc* test for comparison of three or more groups. Two-tailed unpaired Student’s t-test used to compare two groups. *p < 0.05, **p < 0.01.

On day 8 p.i., increased percentages of splenic CD4+ T cells of CD mice showed antigen-specific IFN-γ (Th1) production compared to those of DIO mice (2.4% ± 0.02 vs. 0.7% ± 0.19; p < 0.0001), whereas similar percentages of CD4+ T cells in both groups generated IL-17α (Th17) (7.1% ± 0.98 vs. 5.6% ± 2.33) in response to antigen stimulation (**Figures 4A–D**) (22, 38). Furthermore, CD mice had greater percentages of CD4+ T cells expressing both IFN-γ and IL-17α (Th1/17) (0.7% ± 0.17 vs. 0.3% ± 0.28; p = 0.013) (**Figure 4E**), a subset that is associated with pathogenicity in EAE (39, 40). CD mice had greater proliferation, marked by increased frequency of Ki67 positive cells in PD-1+ CD4+ T population compared to DIO mice (33.4 ± 1.50 vs. 19.7 ± 3.50; p = 0.0125) (**Figure 4F**).

We then examined whether this impaired antigen-specific T cell response in DIO mice was restricted to CD4+ T cells or MOG responses. Immunizing DIO mice with the CD8-activating xenoantigen chicken ovalbumin produced similar reduced responses, thus suggesting that DIO also affects CD8+ T cell priming. DIO mice displayed impaired priming of naïve CD8+ T cells to SIINFEKL peptide, an antigen of ovalbumin (2.18 × 10^4^ ± 0.71 vs. 0.83 × 10^4^ ± 0.21) as detected by appropriate tetramer-specific responses (**Supplemental Figure 4A**). While the relative frequencies of antigen-specific T cells were similar within the CD8+ T cell population, absolute numbers of antigen-specific CD8+ T cells were lower in DIO mice (**Supplemental Figure 4A**). This was due to total reduction in percentages and absolute numbers of T cell subsets found in the spleen after immunization (CFA alone or CFA with SIINFEKL). Within the CD3 population, the relative CD8 frequencies were not different among the groups; however, the absolute number of total CD8 cells was lower in DIO compared to the CD group after immunization (**Supplemental Figure 4B**). Interestingly, CD3+ T cells and the CD8 subset were not different between the groups at steady state (**Supplemental Figure 4C**). SIINFEKL-specific CD8+ T cells upregulated PD-1 with immunization, indicating recent antigen activation (**Supplemental Figure 4D**). Relative frequencies of SIINFEKL-specific T cells expressing PD-1 were similar between CD and DIO mice (**Supplemental Figure 4D**). These results indicate that DIO impairs the generation of antigen-specific T cells.

Although obesity is associated with a pro-inflammatory state (6), assessment of serum cytokine levels in DIO and CD mice on day 2 post-immunization showed no difference in levels of pro-inflammatory cytokines, IL-6 and TNF-α, or anti-inflammatory cytokine IL-10 (**Supplemental Figures 5A–C**). Levels of the innate cell stimulatory cytokines, granulocyte-macrophage colony-stimulating factor (GM-CSF), granulocyte-colony stimulating factor (G-CSF), and neutrophil-migration chemokine CXCL1 were also similar between DIO and CD mice (**Supplemental Figures 5D–F**) (41–44). SOCS1 and SOCS3 expression, likewise, was upregulated in naïve T cells to a similar degree in both DIO and CD mice (**Supplemental Figure 5G**). Ex-vivo proliferation of T cells in response to mitogen activation were similar in splenocytes isolated from DIO mice that were either immunized with CFA and pertussis toxin or received control PBS injection, indicating that naïve T cells were not unresponsive and produced similar cytokine responses (**Supplemental Figure 5H**). Splenocytes from DIO mice treated with LPS, however, had blunted responses to ConA stimulation indicating a naïve T cell paralysis that was not present with CFA and pertussis toxin stimulation or PBS treatment (**Supplemental Figure 5H**). These results indicate that the impaired generation of antigen-specific T cells was not due to inflammatory cytokine-driven paralysis of naïve T cells that can be found in states of cytokine storms or septic events (45).

### Impaired T Cell Priming in DIO Mice Is Associated With High Levels of PD-L1 on Antigen-Presenting Cells in Secondary Lymphoid Organs

**Figure 5:**
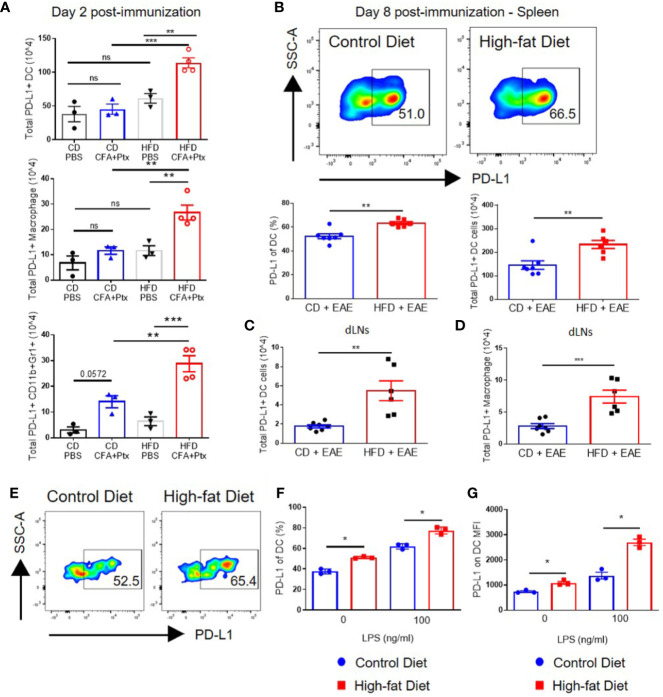
PD-L1 is upregulated on innate cells, including dendritic cells, in DIO mice with activation *in vivo* and *in vitro*. **(A–D)** C57BL/6 male mice were placed on CD or HFD for 6-7 months and immunized subcutaneously with CFA and pertussis toxin on day 0 and 2. SLOs were analyzed on day 2 **(A)** or day 8 **(B–D)** post-immunization. **(A)** Total PD-L1+ dendritic cells (CD11c+F4/80-CD19-CD3-live cells), total PD-L1+ macrophages (F4/80+CD19-CD3-live cells), and total PD-L1+ neutrophils/M1 macrophages (CD11b+GR1+live cells) in the spleens of CD and DIO mice on day 2 post-immunization; n = 3–4/group. **(B)** Representative flow plots of PD-L1 on DCs. Percentages and absolute numbers of DCs expressing PD-L1 in spleens of CD and DIO mice. **(C)** Total PD-L1+ dendritic cells in draining lymph nodes (dLNs). **(D)** Total PD-L1+ macrophages in dLNs of CD and DIO mice; n = 6–7/group, combined from two different experiments. **(E-G)** Splenocytes from C57BL/6 male mice placed on CD or HFD for 6-7 months were cultured *in vitro* with varying concentration of LPS and analyzed by flow cytometry after 20 h. DCs were gated on CD11c+F4/80-CD19-CD3-live cells. **(E)** Representative flow plots of DCs expressing PD-L1 after 1 ng/ml LPS stimulation. **(F)** Percentages of DCs expressing PD-L1 after LPS stimulation. **(G)** MFI of PD-L1 on DC after LPS stimulation; n = 3 technical replicate/LPS concentration, representative of two experiments. Two-tailed unpaired Student’s t-test used to compare two groups. *p < 0.05, **p < 0.01, ***p < 0.001.

Examination of PD-L1 on DCs in spleens and draining lymph nodes on day 2 after CFA and pertussis toxin administration revealed that immunization increased the total numbers of PD-L1-positive DCs in DIO mice to a greater extent than those in CD mice (113.8 × 10^4^ ± 14.8 vs. 45.08 × 10^4^ ± 13.1; p = 0.001) (**Figure 5A**). Similarly, total numbers of PD-L1-positive macrophages and CD11b+Gr1+ cells had greater increases in DIO mice compared to control mice (p = 0.0082 and p = 0.0005, respectively) (**Figure 5A**) (27). CD11b+Gr1+ cells include neutrophils and monocytes/M1 macrophages [Gr1 detects both Ly6G (neutrophils) and Ly6C (present in low levels in neutrophils and in high levels in M1 macrophages)]; however, PD-L1 expression on CD11b+Gr1+ cells is associated with MDSCs (46–48), which have been linked with impaired T cell responses (49). PD-L1 expression on myeloid cells was not different between DIO and CD mice at steady state (after PBS injection in **Figure 5A** and **Supplemental Figure 6**), suggesting that obesity-mediated impairment of PD-L1 upregulation occurs only after activation. This is in agreement with a previous study showing that PD-L1 expression on blood myeloid cells was not upregulated due to obesity in either steady state or in tumor-bearing mice. However, PD-L1 was upregulated in tumor-infiltrating myeloid cells due to obesity (27).

On day 8 after MOG-immunization, the percentages and total numbers of PD-L1-positive DCs were statistically significantly higher in spleens of DIO mice compared to control mice (23.3 × 10^5^ ± 4.2 vs. 14.5 × 10^5^ ± 4.8; p = 0.0053) (**Figure 5B**). Total numbers of PD-L1-positive DCs (p = 0.0028) and macrophages (p = 0.0009) were also statistically significantly higher in draining lymph nodes (dLNs) of DIO mice (**Figures 5C, D**). To assess if PD-L1 upregulation on DCs in DIO mice could be recapitulated *in vitro*, splenocytes from CD and DIO mice were stimulated with LPS for 20 h (50). A higher percentage of DCs from DIO mice expressed PD-L1 compared to CD mice after a 20-h culture even in the absence of LPS (p = 0.000272 for 0 ng/ml of LPS and p = 0.00354 for the 100 ng/ml LPS). DCs from DIO mice had greater PD-L1 upregulation mean fluorescence intensity (MFI) compared to DCs from CD mice after *in vitro* LPS stimulation: the DIO group had a 2.48-fold increase (p = 0.000305) while the CD group had a 1.86-fold increase (p = 0.009807). The difference in MFI between the CD and DIO groups was statistically significant (p = 0.0068 for the 0 ng/ml LPS and p = 0.00191 for the 100 ng/ml LPS) (**Figures 5E–G**). Gating strategy for PD-L1 expression on myeloid cells in shown in **Supplemental Figure 7**. Collectively, these results indicate that in the context of EAE, impaired priming in DIO mice is correlated with increased PD-L1 expression on APCs in secondary lymphoid organs.

### PD-1 Blockade During Priming Increases Antigen-Specific T Cell Generation and Restores EAE Induction and Severity in DIO Mice

**Figure 6:**
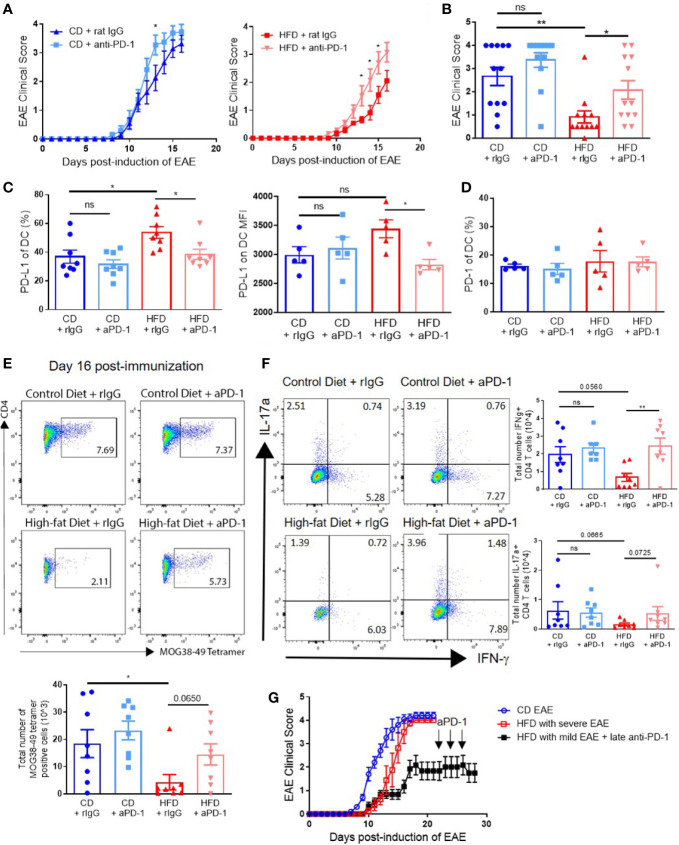
PD-1 blockade during priming restores EAE clinical course in DIO mice. **(A-G)** C57BL/6 male mice were placed on CD or HFD for 6-7 months and immunized subcutaneously with 300 μg **(A-F)** or 100 μg **(G)** MOG35-55 peptide in 5 mg/ml HKMT CFA and 200ng pertussis toxin on day 0 and 2 p.i. For PD-1 blockade, the treatment schedule was 500 μg on day 0 and 250 μg on days 2, 4, 6, 8, and 10 p.i. **(A-F)**, or 500 μg on day 22 and 250 μg on days 24 and 26 p.i. **(G)**. **(A)** EAE clinical score of CD and DIO mice treated with anti-PD-1 or control rat IgG. **(B)** EAE clinical score on day 14 p.i. from A. **(C)** Percentages of DCs expressing PD-L1 and MFI of PD-L1 on DCs in spleen on day 16 p.i. **(D)** Percentages of DCs expressing PD-1 in spleen on day 16 p.i. **(E)** Representative flow plots and numbers of MOG38-49 tetramer staining of CD4+ T cells in CNS on day 16 p.i. **(F)** Representative flow plots and numbers of CD4+ T cells expressing IFN-γ and IL-17α in the CNS on day 16 p.i. **(G)** EAE clinical score of DIO mice that did not initially develop clinical disease treated with anti–PD-1 during chronic phase. EAE clinical scores and bar graphs depict means ± SEM. **(A-E)** Sample size n = 8/group and is combined from two different experiments. **(G)** Sample size n = 4–10/group and is representative of three different experiments. Significance for differences in clinical scores was determined by Mann-Whitney ranking U test. One-way analysis of variance (ANOVA) with Tukey test. Two-tailed unpaired Student t-test used to compare two groups for cell count data when needed. ns: not significant, *p < 0.05, **p < 0.01.

Blockade of the PD-1/PD-L1/2 pathway has been observed to accelerate disease in young mice (24, 25). Since our data show that impaired T cell generation was linked to increased PD-L1 on DCs in the periphery, we hypothesized that PD-1/PD-L1 blockade during the priming stage would restore EAE clinical course in DIO mice to that of control mice. Anti–PD-1 or control rat IgG was administered every other day starting on day 0 until day 10 p.i. to CD and DIO mice. EAE onset and severity in DIO mice treated with anti–PD-1 antibody was markedly increased to an extent comparable to that of CD mice (**Figures 6A, B**). Anti–PD-1 treatment also increased EAE severity in CD mice, reaching significant difference only on day 13 (**Figure 6A**). On day 16 p.i., DIO mice treated with rat IgG had greater percentages of DCs expressing PD-L1 compared to CD mice, but levels of PD-L1 on DCs in DIO mice treated with anti–PD-1 were comparable to those of CD mice treated with anti–PD-1 (**Figure 6C**). Expression of PD-1 on DCs was not different among treatment groups (**Figure 6D**). Importantly, DIO mice treated with anti–PD-1 had greater numbers of MOG-specific T cells in the CNS compared to untreated DIO recipients (**Figure 6E**). Finally, the numbers of IFN-γ-producing CD4+ T cells were statistically significantly increased in DIO mice with PD-1 blockade compared to IgG-treated DIO mice (p = 0.0059) (**Figure 6F**). PD-1 blockade modestly increased the numbers of IL-17α-producing CD4+ T cells (**Figure 6F**). As a number of DIO mice immunized with EAE did not develop severe clinical disease (**Figure 1** and **Supplemental Figure 1**), we tested whether PD-1 blockade during established clinical disease would increase EAE severity, and DIO mice with poor EAE induction did not have increased EAE severity with anti–PD-1 treatment starting on day 22 (**Figure 6G**). These results demonstrate that anti–PD-1 during priming could reverse the impaired responses in DIO mice and restore EAE onset and severity, and underline the critical importance of the PD-1/PD-L1 pathway on the priming stage of this EAE model in obesity.

## Discussion

In this study, by employing the mouse model of autoimmune neuroinflammation, EAE, we observed that establishment of diet-induced obesity in adulthood markedly impairs the priming of autoreactive antigen-specific T cells in secondary lymphoid organs. This resulted in decreased infiltration of the CNS by peripheral immune cells and milder EAE clinical course in DIO mice compared to CD mice. The impairment of T cell responses after immunization was correlated with enhanced upregulation of PD-L1 expression on APCs in DIO mice compared to CD mice. Blockade of the PD-1/PD-L1 pathway during the priming period (day 0–10 p.i.) restored EAE clinical course in DIO mice to similar levels (in both kinetics and severity) to those of CD mice. Interestingly, blockade of this pathway after clinical disease onset had no effect on disease progression in DIO mice that had failed to develop severe EAE. CD mice had only modest increase in EAE severity with anti–PD-1; this may in part be attributed to the robust immunization regimen we employed, which consistently resulted in severe EAE in CD mice. Interestingly, DIO mice had impaired generation of Th1 cells, a subset that was previously shown to facilitate the migration of immune cells into the CNS (51) and Th1/17 cells, a subset that has been associated with pathogenicity in EAE (39, 40). Altogether, our observations are in agreement with previous work indicating that genetic deletion of PD-L1 on DCs or inhibition of CD80 *cis* binding to PD-L1 increases antigen-specific T cell responses (20, 23). Previous studies suggest that the PD-1/PD-L1 pathway promotes tolerance by limiting T cell-DC interactions (52). Thus, blockade of this pathway may promote autoimmunity by prolonging T cell-DC interactions (52).

Several host factors impact the regulation of PD-1/PD-L1 pathway and responsiveness to PD-1/PD-L1 blockade, including age (53), sex (28), BMI (11, 28, 54), and microbiome (55–58). Recent studies have shown obesity-mediated upregulation of the PD-1/PD-L1 pathway in the tumor microenvironment (11, 27). Dendritic cells derived from monocytes in adipocyte-conditioned media from healthy donors with high BMI had greater upregulation of PD-L1 compared to those derived from lean donors (59). The efficacy of PD-1/PD-L1 targeting, particularly in human cancer, has been understood to be primarily relevant on effector T cells in target tissues. Our data further suggest a previously unidentified but important role of PD-1/PD-L1 in secondary lymphoid organs and during the priming of effector T cells in obesity. Interestingly, Yost et al., using single cell RNAseq and T cell receptor sequencing showed that anti-PD-1 treatment promotes antigen spreading by clonal replacement rather than reinvigoration of exhausted tumor specific T cells clones (60). Although Yost et al. did not address the effect of obesity, it is likely that both antigen spreading and priming of T cells in secondary lymphoid organs may be affected by anti-PD1 treatment in our model.

Our results are at variance with previously published studies showing that HFD mice developed more severe EAE compared to CD mice, in part due to increased Th17 responses (29, 30, 61, 62). We initially hypothesized that this may be due to differences in duration of specialized diets, since mice in our study were placed on HFD or CD for 6 months before EAE induction, while in previous studies mice were placed on HFD for either 3 weeks (62) or 10 weeks (29, 30, 61). When we placed 6-week-old mice on HFD or CD for 10 weeks before EAE induction, our DIO mice again showed delayed onset of clinical deficits compared to CD mice. Another deviation between our study and previous studies is the amount of CFA used to induce EAE. Low amounts of mycobacterium and antigen lead to a milder EAE clinical course (63) reminiscent of the clinical course observed in previous studies addressing the effect of HFD in EAE (29, 30). However, when we immunized mice, that were maintained on HFD for at least 6 months, with lower levels of either mycobacterium or MOG peptide we did not observe any differences in the outcome.

Interestingly, in all previous studies addressing the role of DIO in EAE, the kinetics of EAE onset and clinical severity were variable, and mice on CD usually developed a rather mild EAE course. In our experiments, using a robust immunization protocol, CD mice consistently developed clinical symptoms by day 8–9 p.i. and reached peak severity by day 16 p.i., while clinical symptoms in DIO mice appeared on day 11–12 p.i. and reached maximum severity by day 18–21 p.i. In agreement with our data, Hasan et al. showed that DIO mice exhibited milder EAE severity at the early stages but developed more severe EAE clinical symptoms only after day 22 p.i. (61). Though mice used in our studies were significantly older than in previous studies, 8-month-old mice fed CD for at least 6 months had similar EAE clinical course as 8-week-old mice on standard chow, suggesting that the observed differences in DIO mice are not driven by age at the time of EAE immunization. All the above would suggest that factors other than obesity may be responsible for these discrepancies, including genetic drift in strains, differences in vendor source, microbiome, and other environmental factors.

Population-based case studies in Sweden have correlated obese or overweight status in adolescence/young adulthood with increasing risk of MS (64). Similar findings were found in a case-control study with a population in Norway, but no association was found with a population in Italy (65). Stronger associations with childhood obesity have been found for risk of development of clinical disease or poorer response to first-line therapy in pediatric MS patients (66, 67). It is unclear, however, if obesity initiating or persisting in adulthood is associated with increasing pathology and severity in MS as BMI was not correlated with severity in adults (68, 69). The strong correlation of MS severity in human adolescence but not adulthood is interesting and would indicate that both obesity and the age of the immune system can modulate severity to autoimmune diseases.

Our results may have implications in other diseases, such as cancer and infection, where obesity has been shown to impair adaptive immune responses (70–74). It is possible that some of the reported anti-tumor effects observed with PD-1 blockade in obese mice may be due to enhanced naïve T cell priming in the secondary lymphoid organs and not only the effector function in target tissues. Obesity has been associated with impaired T cell adaptive immune responses during viral infection and vaccination that may be improved with early PD-1 blockade during priming (7, 12, 71). In agreement, PD-1 blockade during the initial priming stage enhanced viral control in young, lean mice infected with lymphocytic choriomeningitis virus (LCMV), but did not appear to affect total number of LCMV-specific T cells (17). Given the complexity of obesity, it is possible that several other factors may have contributed to priming impairment and/or PD-L1 upregulation observed. Lymphatic dysfunction in obesity may regulate antigen drainage (75). In addition, other immunosuppressive pathways (i.e., Tregs or MDSCs), also reported increased in obesity, may contribute to the T cell priming defects.

Obesity-induced upregulation of the PD-1/PD-L1 pathway may indicate a greater responsiveness of the immune system to PD-1 blockade. This suggests that obesity could be associated with greater incidence of immune-related adverse events (irAEs) and autoimmunity. Clinical evaluation of irAE incidence and BMI in cancer patient cohorts treated with PD-1 blockade showed either no correlation or positive correlation between the two factors (11, 54, 76, 77). Further studies must be conducted to expand on these conflicting findings as there are several comorbidities associated with obesity and controversy as to whether assessment of obesity is best measured by BMI. Factors that promote immunosuppressive mechanisms, particularly that of PD-1/PD-L1 pathway during adaptive immune responses in settings of obesity or HFD, remain to be elucidated, and better understanding of the mechanism by which obesity impairs priming though the PD-1/PD-L1 pathway will shed important light on cancer and infection management in obese patients. Future studies in the additive or synergistic role of PD-1 blockade with obesity need to be conducted to address issues of autoimmune toxicity that could result from checkpoint blockade.

## Data Availability Statement

The original contributions presented in the study are included in the article/**Supplementary Material**; further inquiries can be directed to the corresponding authors.

## Ethics Statement

The animal study was reviewed and approved by IACUC University of California, Davis.

## Author Contributions

CL, WM, and AS designed research. CL, LK, CD, SC, AN, MW, LV, KS, and AS performed research. CL and AS analyzed data. CL, WM, AS, BB, and AM wrote the paper. All authors contributed to the article and approved the submitted version.

## Funding

This work was funded by National Institutes of Health (grant numbers NIH RO1 CA214048, NIH RO1 CA095572, and NIH RO1 HL56067), by the National Center for Advancing Translational Sciences, National Institutes of Health (grant number UL1 TR001860 and linked award TL1 TR001861), and Shriners Hospitals for Children—Northern California (grant number: 85114-NCA-18). The content is solely the responsibility of the authors and does not necessarily represent the official views of the NIH. Flow cytometry core was supported by the UC Davis Comprehensive Cancer Center Support Grant (CCSG) (grant number P30 CA093373).

## Conflict of Interest

The authors declare that the research was conducted in the absence of any commercial or financial relationships that could be construed as a potential conflict of interest.

## Supplementary Material

Supplementary Figure 1 Diet-induced obesity delays the onset and dampens the severity of clinical symptoms of EAE. **(A)** Non-fasting glucose levels and percentage of HgA1c in blood of C57BL/6 male mice placed on CD or HFD for 6–7 months; n = 3/group. **(B, C)** C57BL/6 male mice were placed on CD or HFD for 6–7 months and induced with EAE; n = 13–14/group **(B)** EAE clinical scores of individual mice. **(C)** Survival curve. **(D)** Clinical scores of C57BL/6 male mice placed on CD or HFD for 6-7 months and immunized subcutaneously with 100 µg MOG35-55 peptide in 5 mg/ml HKMT CFA and 200ng pertussis toxin on day 0 and 2 p.i.; n = 5/group. **(E)** Clinical scores of C57BL/6 male mice placed on CD or HFD for 6–7 months and immunized subcutaneously with 300 µg MOG35-55 peptide in 1 mg/ml HKMT CFA and 200ng pertussis toxin on day 0 and 2 p.i.; n = 3–4/group. **(F)** EAE clinical scores of C57BL/6 male mice placed on CD for 6–7 months or on standard chow only for 8 weeks before EAE induction; n = 3–4/group. **(G)** Individual and pooled EAE clinical scores of 4-month-old DIO and control mice placed on CD or HFD for 10 weeks before EAE induction; n = 4–6/group. Clinical scores are presented as means ± SD. Significance for differences in clinical scores was determined by Mann-Whitney ranking U test. *p < 0.05, ***p < 0.001, ****p < 0.0001.

Supplementary Figure 2 Quantitative PCR analysis for *IFNG* and *IL17A* in the spinal cord reflects delayed induction in DIO mice. **(A, B)** C57BL/6 male mice were placed on CD or HFD for 6-7 months and induced with EAE. Spinal cords were isolated on day 14 and day 21 p.i. and analyzed by qPCR. **(A)** *IFNG* gene expression fold change from baseline values (healthy control spinal cords) on day 14 and day 21 in control and DIO mice post-EAE immunization. **(B)** *IL17A* gene expression fold change from baseline values on days 14 and 21 in CD and DIO mice post-EAE immunization. Bar graphs depict means ± SEM. Sample size n = 2–3/group.

Supplementary Figure 3 Numbers of CNS infiltrating myeloid cells and activated microglia reflect the delayed EAE clinical onset in DIO mice. **(A-D)** C57BL/6 male mice were placed on CD or HFD for 6-7 months and induced with EAE. Iba1 (white), myelin basic protein (red), and SMI-32 (green) immunoreactivity in **(A)** day 14 p.i. white matter. **(B)** day 14 p.i. gray matter. **(C)** day 21 p.i. white matter. **(D)** day 21 p.i. gray matter of spinal cords of CD and DIO mice. **(E)** Isotype control staining for MHCII and PD-L1 flow cytometry in CNS-isolated myeloid cells.

Supplementary Figure 4 DIO mice have reduced SIINFEKL-specific CD8+ T cells in secondary lymphoid organs after immunization. C57BL/6 male mice were placed on CD or HFD for 6-7 months and immunized subcutaneously with 100 μg SIINFEKL peptide in 5 mg/ml HKMT CFA. SLOs were harvested on day 6 post-immunization. **(A)** Representative flow plots of SINNFEKL tetramer staining on CD8+ T cells. Total numbers and percentages of SINNFEKL tetramer-specific CD8+ T cells. **(B)** Percentages and total numbers of CD3+ T cells and CD8 subsets (CD3-gated) in spleens of CD and DIO mice after immunization with CFA only or with CFA+ SIINFEKL. **(C)** Percentages and total numbers of CD3+ T cells and CD8 subsets (CD3-gated) in spleens of steady state CD and DIO mice **(D)** Representative flow plots and bar graphs of percentage on SIINFEKL tetramer-specific CD8+ T cells expressing PD-1 in dLNs. Sample size n = 3–6/group and is combined from two experiments. Bar graphs depict mean ± SEM. One-way analysis of variance (ANOVA) with Tukey’s *post hoc* test for comparison of three or more groups. *p < 0.05, **p < 0.01, ***p < 0.001, ****p < 0.0001.

Supplementary Figure 5 Impaired priming in DIO mice is not due to pro-inflammatory cytokine-induced upregulation of SOCS3 in naïve T cells. **(A–H)** C57BL/6 male mice were placed on CD or HFD for 6–7 months and immunized subcutaneously with 5 mg/ml HKMT CFA and 200ng pertussis toxin on day 0 and 2 or with LPS at 1.5mg/kg IP. **(A)** IL-6, **(B)** TNF-α, **(C)** IL-10, **(D)** GM-CSF, **(E)** G-CSF, and **(F)** KC concentration in the serum on day 2 post-immunization. **(G)** *SOCS1* and *SOCS3* expression in naïve T cells isolated from spleen on day 2 post-immunization. **(H)** MTT assay with ConA stimulation of splenocytes obtained from DIO mice injected with CFA + Pertussis toxin or LPS 1.5 mg/kg on day 2 post-administration. Sample size n = 3–4/group. Two-way analysis of variance (ANOVA) with Sidak’s multiple comparisons test for comparison of three or more groups over time. One-way analysis of variance (ANOVA) with Tukey’s *post hoc* test for comparison of three or more groups. Two-tailed unpaired Student’s t-test used to compare two groups. *p < 0.05, **p < 0.01, ***p < 0.001.

Supplementary Figure 6 No differences in PD-L1 expression in DCs in steady state. PD-L1 expression in DCs from splenocytes isolated from C57BL/6 male mice previously placed on 10% fat CD or 60% fat HFD for 6–7 months.

Supplementary Figure 7 Flow cytometry gating strategy of PD-L1 expression. Splenocytes were isolated from C57BL/6 male mice previously placed on 10% fat CD or 60% fat HFD for 6–7 months and cultured *in vitro* with varying concentration of LPS and analyzed by flow cytometry after 20 h.
