# Supplementary figures and images for: PD-1 Blockade Reverses Obesity-Mediated T Cell Priming Impairment
Source: Front Immunol. 2020 Oct 29;11:590568. doi: 10.3389/fimmu.2020.590568 (PMC7658608; doi:10.3389/fimmu.2020.590568)

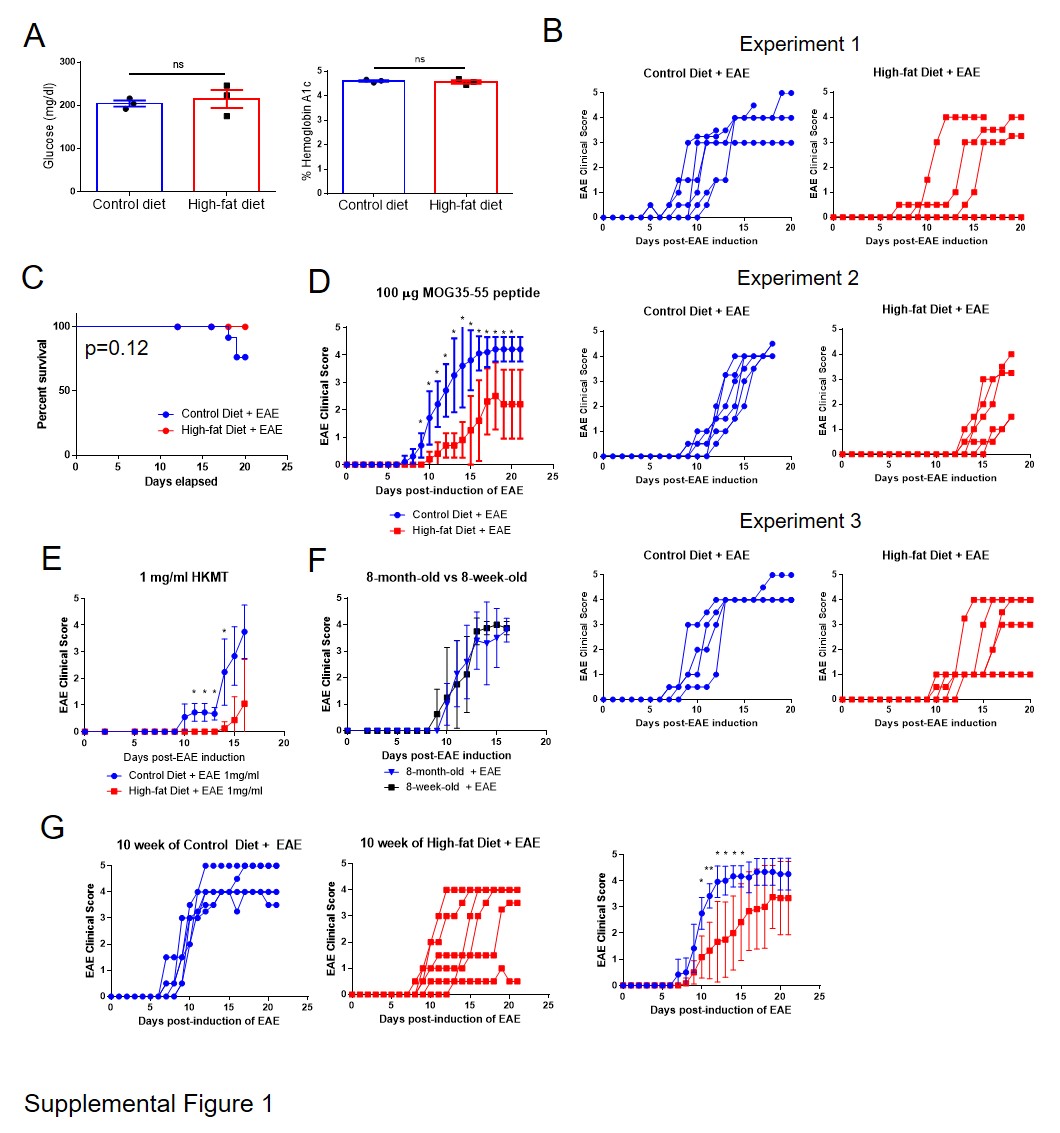

Supplement: Supplementary Figure 1 — Diet-induced obesity delays the onset and dampens the severity of clinical symptoms of EAE. (A) Non-fasting glucose levels and percentage of HgA1c in blood of C57BL/6 male mice placed on CD or HFD for 6–7 months; n = 3/group. (B, C) C57BL/6 male mice were placed on CD or HFD for 6–7 months and induced with EAE; n = 13–14/group (B) EAE clinical scores of individual mice. (C) Survival curve. (D) Clinical scores of C57BL/6 male mice placed on CD or HFD for 6-7 months and immunized subcutaneously with 100 µg MOG35-55 peptide in 5 mg/ml HKMT CFA and 200ng pertussis toxin on day 0 and 2 p.i.; n = 5/group. (E) Clinical scores of C57BL/6 male mice placed on CD or HFD for 6–7 months and immunized subcutaneously with 300 µg MOG35-55 peptide in 1 mg/ml HKMT CFA and 200ng pertussis toxin on day 0 and 2 p.i.; n = 3–4/group. (F) EAE clinical scores of C57BL/6 male mice placed on CD for 6–7 months or on standard chow only for 8 weeks before EAE induction; n = 3–4/group. (G) Individual and pooled EAE clinical scores of 4-month-old DIO and control mice placed on CD or HFD for 10 weeks before EAE induction; n = 4–6/group. Clinical scores are presented as means ± SD. Significance for differences in clinical scores was determined by Mann-Whitney ranking U test. *p < 0.05, ***p < 0.001, ****p < 0.0001. [file Image_1.jpeg]

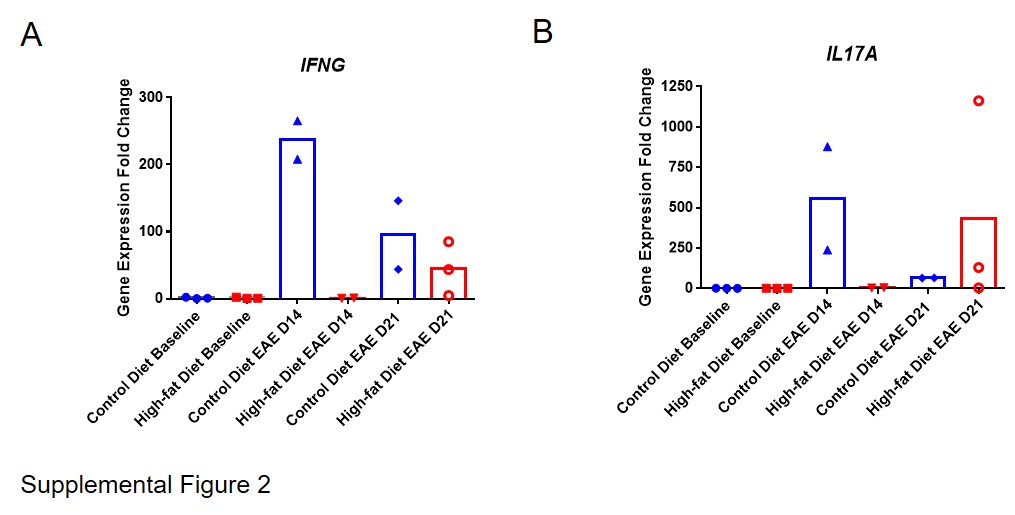

Supplement: Supplementary Figure 2 — Quantitative PCR analysis for IFNG and IL17A in the spinal cord reflects delayed induction in DIO mice. (A, B) C57BL/6 male mice were placed on CD or HFD for 6-7 months and induced with EAE. Spinal cords were isolated on day 14 and day 21 p.i. and analyzed by qPCR. (A) IFNG gene expression fold change from baseline values (healthy control spinal cords) on day 14 and day 21 in control and DIO mice post-EAE immunization. (B) IL17A gene expression fold change from baseline values on days 14 and 21 in CD and DIO mice post-EAE immunization. Bar graphs depict means ± SEM. Sample size n = 2–3/group. [file Image_2.jpeg]

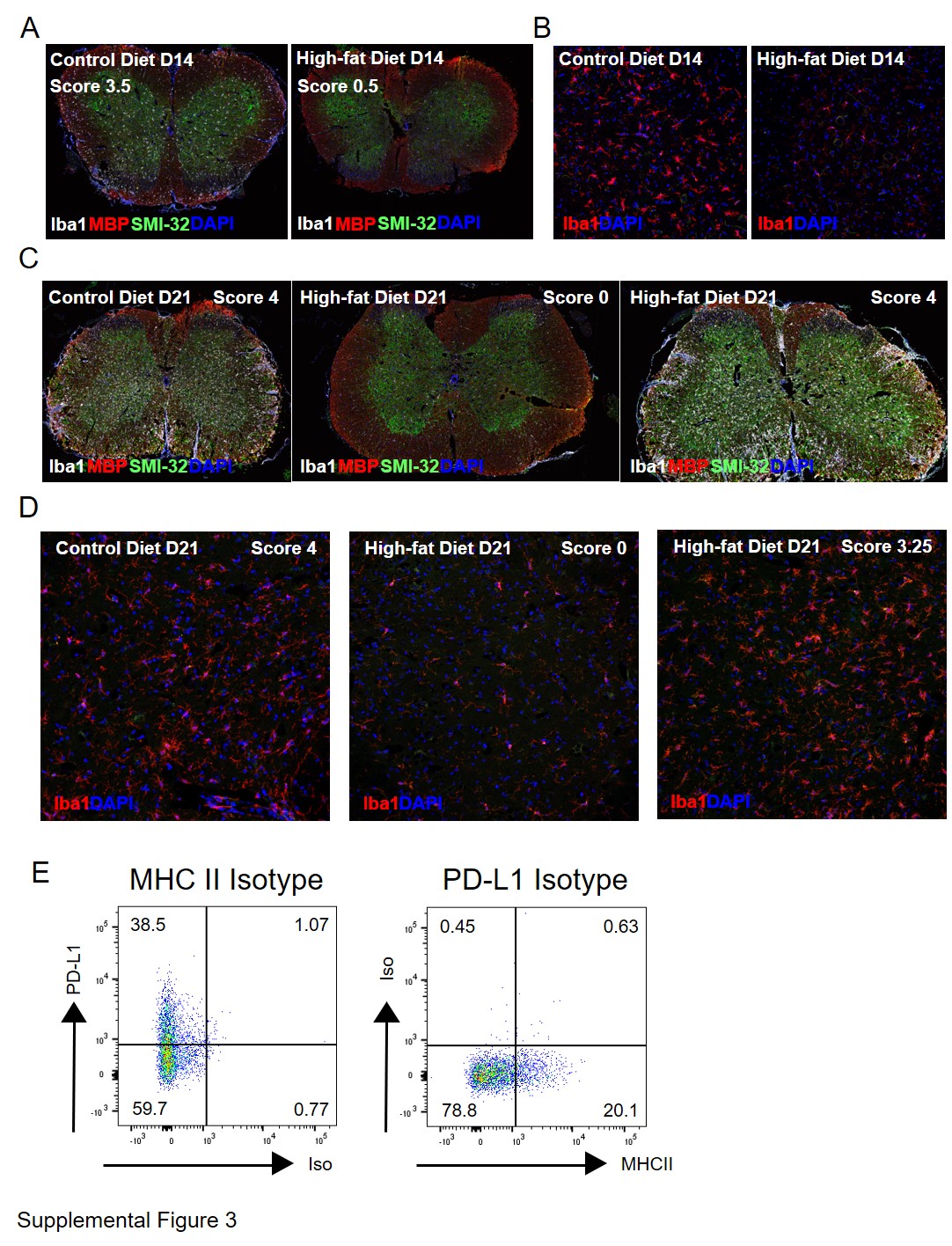

Supplement: Supplementary Figure 3 — Numbers of CNS infiltrating myeloid cells and activated microglia reflect the delayed EAE clinical onset in DIO mice. (A-D) C57BL/6 male mice were placed on CD or HFD for 6-7 months and induced with EAE. Iba1 (white), myelin basic protein (red), and SMI-32 (green) immunoreactivity in (A) day 14 p.i. white matter. (B) day 14 p.i. gray matter. (C) day 21 p.i. white matter. (D) day 21 p.i. gray matter of spinal cords of CD and DIO mice. (E) Isotype control staining for MHCII and PD-L1 flow cytometry in CNS-isolated myeloid cells. [file Image_3.jpeg]

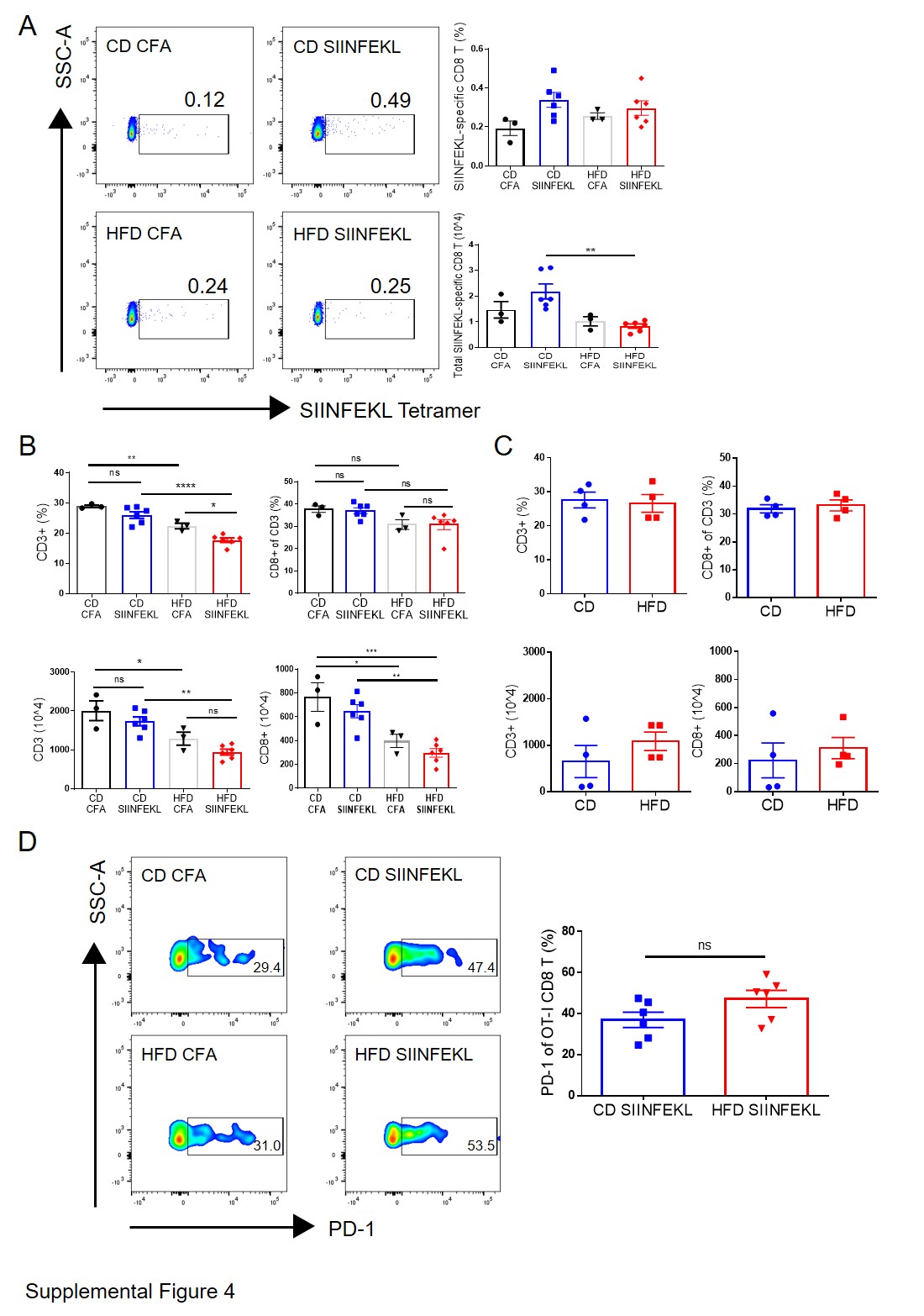

Supplement: Supplementary Figure 4 — DIO mice have reduced SIINFEKL-specific CD8+ T cells in secondary lymphoid organs after immunization. C57BL/6 male mice were placed on CD or HFD for 6-7 months and immunized subcutaneously with 100 μg SIINFEKL peptide in 5 mg/ml HKMT CFA. SLOs were harvested on day 6 post-immunization. (A) Representative flow plots of SINNFEKL tetramer staining on CD8+ T cells. Total numbers and percentages of SINNFEKL tetramer-specific CD8+ T cells. (B) Percentages and total numbers of CD3+ T cells and CD8 subsets (CD3-gated) in spleens of CD and DIO mice after immunization with CFA only or with CFA+ SIINFEKL. (C) Percentages and total numbers of CD3+ T cells and CD8 subsets (CD3-gated) in spleens of steady state CD and DIO mice (D) Representative flow plots and bar graphs of percentage on SIINFEKL tetramer-specific CD8+ T cells expressing PD-1 in dLNs. Sample size n = 3–6/group and is combined from two experiments. Bar graphs depict mean ± SEM. One-way analysis of variance (ANOVA) with Tukey’s post hoc test for comparison of three or more groups. *p < 0.05, **p < 0.01, ***p < 0.001, ****p < 0.0001. [file Image_4.jpeg]

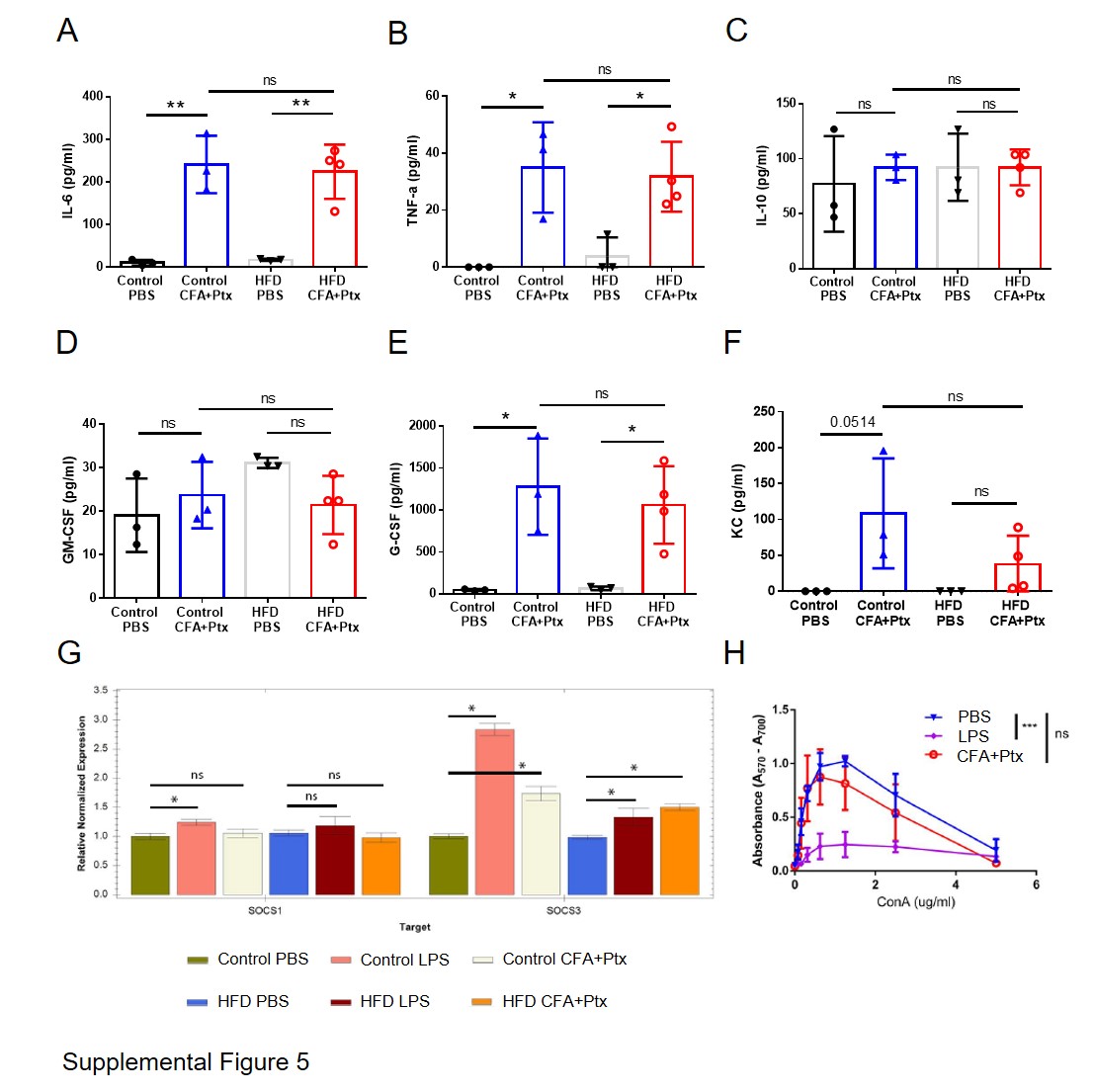

Supplement: Supplementary Figure 5 — Impaired priming in DIO mice is not due to pro-inflammatory cytokine-induced upregulation of SOCS3 in naïve T cells. (A–H) C57BL/6 male mice were placed on CD or HFD for 6–7 months and immunized subcutaneously with 5 mg/ml HKMT CFA and 200ng pertussis toxin on day 0 and 2 or with LPS at 1.5mg/kg IP. (A) IL-6, (B) TNF-α, (C) IL-10, (D) GM-CSF, (E) G-CSF, and (F) KC concentration in the serum on day 2 post-immunization. (G) SOCS1 and SOCS3 expression in naïve T cells isolated from spleen on day 2 post-immunization. (H) MTT assay with ConA stimulation of splenocytes obtained from DIO mice injected with CFA + Pertussis toxin or LPS 1.5 mg/kg on day 2 post-administration. Sample size n = 3–4/group. Two-way analysis of variance (ANOVA) with Sidak’s multiple comparisons test for comparison of three or more groups over time. One-way analysis of variance (ANOVA) with Tukey’s post hoc test for comparison of three or more groups. Two-tailed unpaired Student’s t-test used to compare two groups. *p < 0.05, **p < 0.01, ***p < 0.001. [file Image_5.jpeg]

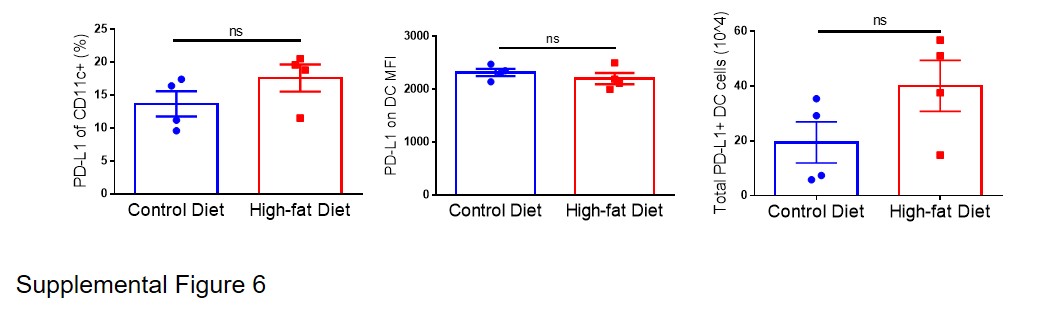

Supplement: Supplementary Figure 6 — No differences in PD-L1 expression in DCs in steady state. PD-L1 expression in DCs from splenocytes isolated from C57BL/6 male mice previously placed on 10% fat CD or 60% fat HFD for 6–7 months. [file Image_6.jpeg]

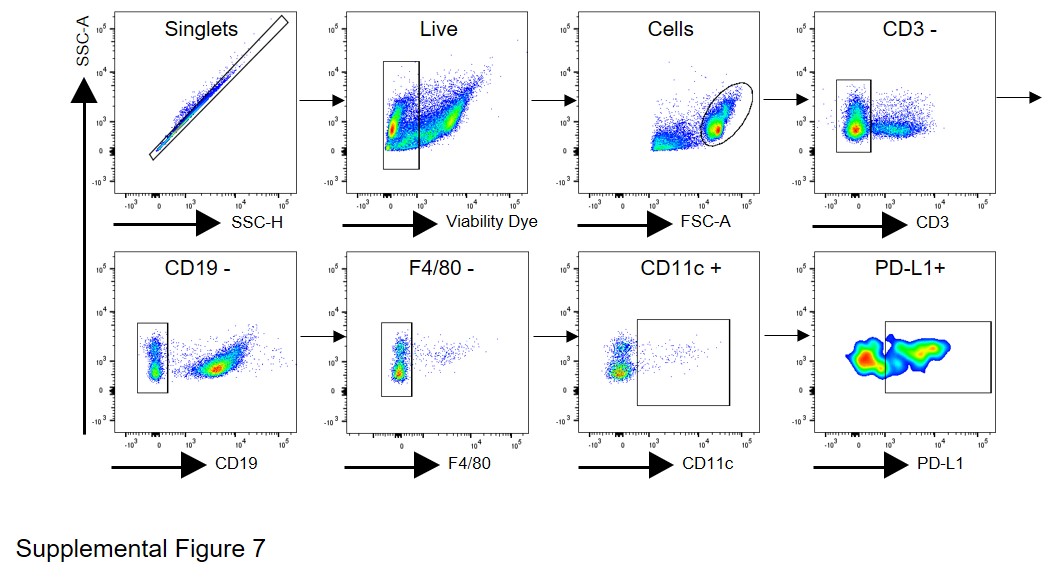

Supplement: Supplementary Figure 7 — Flow cytometry gating strategy of PD-L1 expression. Splenocytes were isolated from C57BL/6 male mice previously placed on 10% fat CD or 60% fat HFD for 6–7 months and cultured in vitro with varying concentration of LPS and analyzed by flow cytometry after 20 h. [file Image_7.jpeg]
